# Supplementary material for: Childhood and adult socioeconomic position interact to predict health in mid life in a cohort of British women
Source: PeerJ. 2017 Jun 29;5:e3528. doi: 10.7717/peerj.3528 (PMC5493971; doi:10.7717/peerj.3528)
Supplement: Supplemental Information 1 [file peerj-05-3528-s001.pdf]

**Supplemental Information for 'Childhood and adult socioeconomic position interact to predict health in mid life in a cohort of British women'**

## S1. Study variables and descriptive statistics

Variables used in the present study, along with their NCDS codes and descriptive statistics, are shown in table S1.

| Variable | NCDS code | Descriptives |
|---|---|---|
| Childhood socioeconomic position (cSEP) | Derived by PCA [see section 2] from: n2397, n492, n49/n46 | Mean 0, s.d. 1 |
| Adult socioeconomic position (aSEP) aged 23 | Derived by PCA [see section S2] from: curroscr, hqual23 | Mean 0, s.d. 1 |
| Adult socioeconomic position (aSEP) aged 33 | Derived by PCA [see section S2] from: curroscr, n540033, hqual23, hqual33 | Mean 0, s.d. 1 |
| Adult socioeconomic position (aSEP) aged 42 | Derived by PCA [see section S2] from: curroscr, n540033, sc, hqual23, hqual33, n7iamt | Mean 0, s.d. 1 |
| Self-rated health aged 23 | n5739 | Mean 3.30, s.d. 0.68 |
| Self-rated health aged 33 | n503913 | Mean 3.17, s.d. 0.70 |
| Self-rated health aged 42 | hlthgen | Mean 3.07, s.d. 0.77 |

Table S1. NCDS codes and descriptive statistics for variables used.

## S2. Derivation of aSEP and cSEP variables by Principal Components Analysis

The cSEP and aSEP variables were produced by saving the first factor (scores derived using the regression method) from a principal components analysis (PCA) of the component variables. Missing values were replaced with the mean. The extracted factors accounted respectively for 56% of the variation (cSEP), 76% of the variation (aSEP aged 23), 69% of the variation (aSEP aged 33), and 57% of the variation (aSEP aged 42). The KMO measures of sampling adequacy were 0.64 (cSEP), 0.50 (aSEP aged 23), 0.70 (aSEP aged 33), and 0.78 (aSEP aged 42); whilst $p < 0.01$ for Bartlett's sphericity test in all cases (cSEP: $\chi^2_3 = 2805.74$; aSEP aged 23: $\chi^2_3 = 2819.78$; aSEP aged 33: $\chi^2_6 = 25162.79$; aSEP aged 42: $\chi^2_{15} = 30642.85$). A KMO measure of at least 0.5 and a Bartlett's test with $p < 0.05$ are considered prerequisites for the suitability of PCA.

Loadings on the derived SEP components were as given in table S2.

| Component | Constituent variable | Loading |
|---|---|---|
| cSEP | Father's social class | -0.76 |
| | Mother's age at leaving education | 0.72 |
| | Proportion of professional fathers in child's school class | 0.76 |
| aSEP aged 23 | Highest educational qualification, age 23 | 0.87 |
| | Social class, age 23 | -0.87 |
| aSEP aged 33 | Highest educational qualification, age 23 | 0.91 |
| | Social class, age 23 | -0.74 |
| | Highest educational qualification, age 33 | 0.92 |
| | Social class, age 33 | -0.73 |
| aSEP aged 42 | Highest educational qualification, age 23 | 0.87 |
| | Social class, age 23 | -0.72 |
| | Highest educational qualification, age 33 | 0.88 |
| | Social class, age 33 | -0.74 |
| | Social class, age 42 | -0.69 |
| | Grouped household income, age 42 | 0.56 |

Table S2. Loadings of individual constituent variables on the adult socioeconomic position (aSEP) and childhood socioeconomic position (cSEP) factors.

The aSEP and cSEP variables were re-standardised in running each statistical model, since different missing cases at each age point mean that the effective mean and standard deviation can otherwise depart slightly from 0 and 1 respectively in particular models.
